# JAK inhibitors in toxic epidermal necrolysis a new frontier in therapeutics

**DOI:** 10.1097/MS9.0000000000004676

**Published:** 2026-01-09

**Authors:** Fatima Tuz Zahra, Areej Imtiaz, Aleeza Khan, Mahnoor Fatima

**Affiliations:** a Nishtar Medical College, Nishtar Medical University Multan, Pakistan; b Shaheed Ziaur Rehman Medical College, Rajshahi Medical University, Bangladesh

Toxic Epidermal Necrolysis (TEN) represents the most severe end of the Stevens Johnson Syndrome (SJS)/TEN spectrum, presenting as a rare yet devastating and unequivocally life-threatening mucocutaneous disorder defined by extensive skin detachment and frequent multi-organ complications. Although its annual incidence is merely 1–2 per million individuals it imposes a disproportionately profound and catastrophic burden despite its rarity[1]. Reported mortality rates remain high, estimated between 20 and 50%, a staggering figure that underscores the lethal nature of the disease, shaped by factors such as total skin detachment and coexisting systemic illnesses[2]. Despite decades of clinical experience, therapeutic approaches to TEN remain heterogeneous and often unsatisfactory. Agents such as intravenous immunoglobulin (IVIG), systemic corticosteroids, cyclosporine, and TNF-α have shown inconsistent efficacy, and consensus on an optimal treatment strategy has not been reached[3]. This absence of consensus not only exposes a critical gap in management but also perpetuates uncertainty in clinical decision-making, thereby compromising patient outcomes. It underscores the urgent need for mechanism-based, evidence-driven, and targeted therapeutic approaches with the genuine potential to transform the grim prognosis of this relentlessly life-threatening disorder. This article was written in accordance with the TITAN guideline checklist 2025[4].

For a disease as lethal as TEN, incremental progress is failure. This disease demands transformation, and JAK inhibitors stand as the first therapy with the power to deliver it. The Janus kinase–signal transducer and activator of transcription (JAK–STAT) pathway is a central mediator of immune activation, mediating downstream effects of cytokines including interferon-γ, interleukin-6, and interleukin-15[5]. In toxic epidermal necrolysis (TEN), overactivation of this pathway fuels cytotoxic T-cell and NK cell–mediated injury, driving keratinocyte apoptosis and large-scale epidermal injury[6]. Agents like tofacitinib, baricitinib, ruxolitinib, and upadacitinib act as JAK inhibitors, targeting intracellular phosphorylation signaling and leading to suppression of the cytokine-amplified inflammation central to disease pathogenesis^[^7,8^].^ Unlike broad and unpredictable immunosuppressants, JAK inhibitors offer a precise, mechanism-driven approach that disrupts key pathological pathways, positioning them as a promising therapeutic option for TEN.

The transition of JAK inhibitors from theoretical mechanisms to clinical practice has occurred with a remarkable speed. Although the published data remain scarce, they consistently show promising outcomes across various therapeutic regimens and patient settings. The most compelling evidence comes from Nordmann *et al*, who described seven patients diagnosed with TEN treated by off-label use of four different JAK inhibitors; tofacitinib, baricitinib, upadacitinib, and abrocitinib. All patients survived at the 30-day mark with no major side effects. Moreover, pSTAT1 staining showed a marked reduction following JAK inhibitor therapy. The cases spanned diverse etiologies, including chemotherapy for small cell lung cancer, checkpoint inhibitor therapy for hepatocellular carcinoma, allopurinol, metronidazole, and fluoroquinolone exposure. SCORTEN scores ranged from 1 to 5, corresponding to predicted mortality rates of 12.9 to >90%. Despite these high predicted risks, patients experienced rapid disease control with cessation of new lesion formation within 48–72 hours and substantial re-epithelialization within 1–2 weeks[8]. These uniformly favorable outcomes point to a potentially paradigm-shifting effect, suggesting JAK inhibition may alter the natural history of TEN in a field where only modest gains have long been the norm.

Another study by Li *et al* reported a case of tacrolimus-induced SJS in which administration of tofacitinib, a JAK inhibitor, produced effective outcomes[9]. Zhou *et al* documented a case of a 54-year-old woman with SJS/TEN overlap. The patient received upadacitinib in combination with methylprednisolone (a corticosteroid). Within days, the disease progression stopped, and the patient achieved complete healing with 100% re-epithelialization. Moreover, methylprednisolone was gradually tapered at a rate of 10 mg per week, with no adverse effects reported during therapy and follow-up[10]. This case underscores the potential complementarity between JAK inhibition and corticosteroids, enabling disease control with reduced cumulative immunosuppressive burden. A study by Tabbakh *et al* reports a case of a 65-year-old female patient with epithelioid pleural mesothelioma. She received four cycles of ipilimumab and nivolumab, immune checkpoint inhibitors, which induced TEN. Such cases are more challenging, as checkpoint inhibitors prime the immune system for heightened toxicity, making traditional treatments with immunosuppressants less effective. Treatment with ruxolitinib resulted in the resolution of skin detachment, accompanied by systemic stabilization[11]. This case is clinically quite significant because checkpoint inhibitor–induced TEN is becoming more common in oncology and has shown poorer outcomes with conventional therapeutic regimens.

The evidence is clear and demands urgent attention. Across different JAK inhibitors, diverse clinical triggers, and varying treatment settings – whether used alone or in combination – the reports show a striking consistency of positive outcomes. This kind of reproducibility is rare in TEN and cannot be ignored. Traditionally, mortality rates for patients with extensive TEN treated with IVIG alone or supportive care plus steroids have been reported at 30–40%[12]. As a result, the 100% survival rate in Nordmann’s cohort and the positive results in other cases are extremely extraordinary and point to a therapeutic signal potent enough to demand immediate formal trials.

One of the most persuasive arguments for JAK inhibitors is not just their efficacy, but their relative safety compared to conventional treatments. Corticosteroids carry risks of sepsis, delayed wound healing and GI bleeding, while therapy with cyclosporine and IVIG might also produce significant adverse effects. In contrast to this, JAKi produces no severe side effects to date[8]. In the context of the significant survival benefit, the lack of significant toxicity is not a coincidental discovery; rather, it is a therapeutic signal of critical importance. Rather than causing global immunosuppression, JAK blockade interrupts a specific cytokine-driven inflammatory cascade central to keratinocyte apoptosis. The implications are profound: we might finally have a treatment that saves lives without requiring the high systemic costs that have long been associated with management of TEN.

A great deal of interest has been sparked by the early reports of positive results, and this has now translated into the first prospective investigations. Particularly noteworthy are two clinical trials. Tofacitinib alone is being tested in patients with SJS/TEN in the monotherapy trial (Taiwan), with endpoints including ocular complications, mortality, and re-epithelialization rate[13]. In China, a multicenter combination trial is assessing methylprednisolone in addition to a JAK inhibitor in 30 patients; it is anticipated to be completed in 2026. This trial will help determine whether JAK inhibitors can eventually replace corticosteroids entirely or if they function best as adjuncts to them (with potential steroid-sparing benefits)[14]. Clinicians continue to follow case-based evidence and mechanistic rationale until results are obtained. However, the very fact that these trials are taking place indicates a paradigm shift: JAK inhibitors are moving from being experimental agents on the fringe to being evaluated in a systematic manner.

Emerging evidence suggests that JAK inhibitors may represent one of the most promising advances in the management of toxic epidermal necrolysis. Unlike traditional therapies, they target the disease at its core, offering a mechanism-based strategy where conventional approaches so often come up short. Early reports are encouraging, showing not only the ability to halt disease progression and speed re-epithelialization, but also a safety profile that appears more favorable than long-standing immunosuppressive regimens.

What the field needs now is not caution, but conviction. Large, collaborative trials and international registries must move quickly to clarify how best to use these drugs-the right timing, the right dose, and whether they should stand alone or work alongside steroids. If the early signals hold true, JAK inhibitors could move TEN treatment from trial and error to precision medicine, with the potential to finally change the survival curve in one of dermatology’s most lethal conditions.

## Data Availability

Not applicable.
